# Contagious Pleuro-Pneumonia

**Published:** 1886-10

**Authors:** 


### CONTAGIOUS PLEURO-PNEUMONIA.

An eminent veterinarian stated at a public meeting a year ago, that the people of the United States would never waken up to the necessity of proper legislation for the suppression of contagious diseases among our domestic animals, until the Russian Rinderpest is imported and sweeps off two million cattle in as many months. It is to be hoped that the present outbreak of contageous pleuro-pneumonia in Chicago may prove a sufficient lesson; the lung plague is a scourge severe enough in the United States to day, without asking for more. The veterinarians of the Eastern States have appealed to the Western stock raisers to protect themselves, and individually the latter have acknowledged the importance of strong measures, but have not had the organization to compete in the political arena with the millionaire butchers of Chicago and the large cities.

A comparison of the cattle statistics of several of the infected Eastern states, with some of the important cattle states, which are threatened in the West, will show plainly the difference between them, of interest in self-protection.

In 1880* there were in

| | MILCH COWS. | | OXEN AND OTHER CATTLE. | |
|---|---|---|---|---|
| Pennsylvania | 828,333 | | 626,525 | |
| New York | 1,431,700 | | 648,542 | |
| New Jersey | 153,700 | | 84,500 | |
| | | 2,413,733 | | 1,359,567 |
| Illinois | 709,308 | | 1,222,947 | |
| Iowa | 782,460 | | 1,411,512 | |
| Missouri | 542,295 | | 1,697,749 | |
| | | 2,034,063 | | 4,332,208 |

In the three Eastern States where the lung plague has been a permanent fixture for a third of a century, the milch cow exists in double numbers over the ox and other cattle; all cattle are kept in dairies or on farms with their movements among other cattle approximately controlled. The current of trade is toward these states, and the ultimate destination of nearly all these cattle is the slaughter house. In the West the milch cow and isolated cattle are but as 1 to 2, in comparison with the steers and other cattle; the animals are not individually under the eyes of the owner and constant communication is kept up among all cattle, as they pass from the hands of the breeder to those of the fattener, or in their migrations for better pasture. Contagion in the Eastern States is kept smouldering out of sight and might readily be extinguished, as it was in Massachusetts for only a little over $77,000, were it not for the penny wise policy of the state governments. Contagion in the Western states is to day an ember that a small pile of gold will smother out. Once let the blaze get beyond the dairies and distilleries of Chicago, (which resemble in principle, the condition of cattle in the Atlantic States), and the $300,000,000 worth of cattle in the West will be reduced to keep company with the cattle trade of Australia and the Cape of Good Hope.

The *Drover's Journal* of Chicago, presumably the organ of the beef exporters, has been, since the recent investigation in Chicago, maliciously puerile, in quoting the reported "pleuro-

*The increase in the United States, of horned cattle from 1870 to 1880, was 12,000,000. Since 1880 it has probably been 20,000,000.

pneumonia racket," stating that the sick cattle are only suffering from ordinary pneumonia, produced by the bad hygienic surroundings of distilleries, etc.; one would suppose from some of the statements made, that Dr. Salmon and his profession had a grudge against the trade of the West. It certainly is unfortunate that many persons have a prejudice against meat of animals slaughtered by law, and are unwilling to accept the century's experience of European cities, that has demonstrated that beef from cattle which have had pleuro-pneumonia can be eaten with impunity, when inspection shows that it is free from fever or purulent taint. The history furnished us so far has been too meager to determine exactly the extent of infection in Illinois, and what may be the result of the investigations and appliance of the limited means at the disposal of the government authorities. We trust that by our next issue a definite result will have been reached, in the mean time it is the duty of all to instruct our legislators as to the only panacea of this scourge—*extermination—the pole axe for all cattle affected with or exposed to the contagion of contagious pleuro-pneumonia, or the alternative, the ruin of the cattle trade of the West.* H.

---

ELEPHANT'S BRAIN.—The brain of the elephant which was removed by Doctors Spitzka and Brill on the occasion of the slaughter of that animal at Central Park, weighed thirteen pounds and two ounces. Each cerebral hemisphere, singly, weighed four pounds and twelve ounces. The weight of the mesencephalon, with attached optic nerve origin of the pons oblongata and cerebellum (*or ambitus*) together, was three pounds and ten ounces. If we are not mistaken this is the largest brain ever weighed and the precaution has been taken to ensure correctness by weighing it twice. Dr. Spitzka was enabled to confirm on this occasion, the absence of the true pyramids in the elephant. He says "the representation in the text book of the base of the elephant's brain is a caricature. The olivary bodies are extraordinarily prominent, and they project in relief beyond the inter-olivary field, which is formed by two slender columns, parallel to each other and not apparently converging at the spinal end of the oblongata. The deep trench between the pons and olives is not filled out, as in the porpoise by a trapezium, it is therefore in the following animals alone, that the trapezium is completely hidden from view by the redundant development of the pons: man, anthropoid apes and elephant."

A detailed description of the most interesting features of the elephant's brain is promised us.

The gun shot wound which killed the animal, had fortunately injured the brain in the least important district, namely the apex of one temporal lobe (the right). The brain was extensively larcerated here in a cubic area of two inches each way, by fragments of bone which the shot had driven before it. A large hemorrhage had ensued completely injecting the arachnoid sac over the right cerebral hemisphere, the base of the brain, the epi-cerebellar space and the upper part of the spinal canal. The brain weights above given, did not therefor include the membranes which had to be separated to remove the clots. It is intimated to us, that the deep structure of the elephant's cerebral hemispheres indicate a remarkable approximation to the type of the porpoise. As is well known, the cetacea and land animals near the elephant in zoological position, agree in placentation as well as in other important points. C.

---

DEFECTS OF THE PRESENT U. S. ARMY VETERINARY SYSTEM. —It is to be deplored that the position of veterinary surgeon in the U. S. Army, is anything but a desirable one. Without proper rank and sufficient pay, he cannot command respect nor the obedience necessary to the carrying out of his orders and he is debarred the society of the officers. In European armies, on the contrary, he is a commissioned officer, ranking as high as colonel.

A law to regulate the matter has been drafted and submitted to Congress which, if passed, will improve the status of the veterinarians, prove a source of economy to the service and protection to army animals. C.

---

HOMOGLOBINURIA.—A late number of *La Clinica Veterinario*, contains an excellent article on this interesting subject and we promise our readers a translation of it in the next number of the JOURNAL.

---

ALKATRITS—Stearns & Co., of Detroit, have placed in the market a preparation with the above name which enables physicians or dispensers, to administer the potent alkaloids without encountering the difficulties and dangers hitherto found on account of the extremely minute doses necessary.

---

TO OUR READERS AND EXCHANGES.—Beginning with this number, THE JOURNAL OF COMPARATIVE MEDICINE AND SURGERY is issued from the office of Dr. A. L. Hummel, publisher, 1217 Filbert street, Philadelphia, Pa. Address all business communications to him but send exchanges and articles for publication to the editors, care of Central Park Menagerie, New York City.

